# ‘A successful re-blocking project is when the government works hand in hand with the community’: Factors determining the longevity of re-blocking projects for fire-risk reduction

**DOI:** 10.4102/jamba.v18i1.2017

**Published:** 2026-02-25

**Authors:** Siyachuma S. Sintu, Robyn Pharoah

**Affiliations:** 1 Research Alliance for Disaster and Risk Reduction, Faculty of Arts and Social Sciences, Stellenbosch University, Stellenbosch, South Africa

**Keywords:** re-blocking, informal settlements, fire, risk reduction, Cape Town

## Abstract

Informal settlements are highly vulnerable to fires, due to housing materials, poor infrastructure, dangerous energy sources and housing densities that facilitate the spread of fires and hamper firefighting. Re-blocking aims to reduce fire risk by spatially reconfiguring and de-densifying settlements and upgrading services. However, re-blocked settlements often re-densify, potentially undermining long-term risk reduction benefits. Using case studies of two re-blocked informal settlements, this study aims to explore informal settlement dwellers’ perspectives on re-blocking interventions and identify the factors influencing their long-term success, especially the dynamics leading to re-densification. A qualitative research approach was used, employing a comparative case study design. Data were collected through 10 in-depth semi-structured key-informant interviews with officials, implementers and community leaders, and six focus groups (three in each community) involving 84 participants selected based on involvement in the process. The findings suggest that the primary reason for re-densification was that the allocated housing spaces were too small for households’ needs or growth. Others included opportunism and push for better housing conditions. Although participation was intended, some residents felt the process was rushed or exclusive and that decision-making neglected their needs, leading to dissatisfaction that could reduce the longevity of spatial changes. The findings suggest the long-term effectiveness hinges on addressing beneficiaries’ core needs and expectations. Ultimately, deep, inclusive and unrushed community engagement is crucial to capture diverse needs, ensure sustainable outcomes and achieve intended risk reduction benefits.

**Contribution:**

This research fills a practical gap by examining the underlying non-fire safety factors that drive re-densification and provide insight into how re-blocking interventions can be improved.

## Introduction

The world is urbanising rapidly. The United Nations Human Settlements Programme (UN Habitat) estimates that the proportion of people living in urban areas will increase from 56% in 2021 to 68% by 2050 (UN Habitat 2022) – amounting to an increase of 2.2 billion people, primarily in Africa and Asia. Many of those living in, and migrating to, towns and cities live in informal settlements. As many as 20% of the world’s population, or 1.6 billion people, lived in inadequate housing in 2022, 1 billion of whom resided in informal settlements, primarily in urban areas (UN Habitat 2022). Informal settlements are defined as dwellings located on land that is not declared, designed or surveyed as a place where people can live. The proliferation of informal settlements is linked to a range of urban dynamics, such as rapid urbanisation, poverty and poor urban planning, inadequate affordable housing, as well as spatial and economic marginalisation (Walls 2020).

Informal settlements are a ubiquitous feature of South Africa’s towns and cities. South Africa’s General Household Survey found that 12.2% of the population were living in informal settlements in 2023 (Statistics South Africa 2023), with over 2 million people living in 4297 informal settlements across the country in 2023, most of which were concentrated in the major metropolitan areas of Johannesburg (210), Cape Town (464) and eThekwini (530) (Comins 2023).

These settlements are usually characterised by severe poverty, overcrowding and inadequate services (Marutlulle 2019) and are prone to hazards such as fires, floods, diseases, pollution, environmental degradation and extreme heat (Marutlulle 2019). Fires are among the most common hazards in informal settlements (Gibson, Engelbrecht & Rush 2019). Causes of fires include electrical faults, human actions and natural events such as wildfires spreading into peripheral settlements (Quiroz, Walls & Cicione 2021).

Several features of informal settlements contribute to the risk of fires. Many people depend on dangerous energy sources such as candles, paraffin and informal electricity connections, which can ignite fires (Adelekan et al. 2015; Pharoah et al. 2022; Quiroz et al. 2021). Dwellings are usually built of flammable materials such as wood, cardboard and plastic sheeting, while inadequate service provision can lead to the accumulation of flammable refuse between dwellings (Williams et al. 2019). Poor road access and inadequate infrastructure and services, including water infrastructure and fire hydrants, makes it difficult to reach fires and hampers the operation of firefighting services (Walls et al. 2020). The marginality of informal settlements also reduces preparedness and risk reduction in these communities (Abunyewah, Gajendran & Maund 2018) as people do not have the money to spend on physical changes to reduce or avoid the possible effects of hazards.

Cape Town has been recognised as a fire hotspot and one of the country’s most fire-prone cities (Pharoah et al. 2022; Walls et al. 2020). In 2023, for instance, the City of Cape Town’s fire services reported 2416 informal settlement fires (compared to 1683 in formal dwellings) (FPASA 2023). Most fires damage and destroy a small number of dwellings, but they can burn through whole settlements. In January 2013, for example, a fire in Khayelitsha, one of Cape Town’s largest low-income areas, left five dead and 4000 homeless (Sacks 2013). In 2017, a fire in another large informal settlement, Imizamo Yethu, affected approximately 10 000 people (Kahanji, Walls & Cicione 2019), while a fire in a settlement named Masiphumele, in November 2020, left two dead and displaced 4000 (Pitt & O’Regan 2020). In addition to deaths and injuries, such fires further impoverish already vulnerable households (Pharoah 2009; Raphela 2011; Rush et al. 2020; Twigg et al. 2017; Walls et al. 2019c), highlighting an urgent need for measures to reduce the frequency of fires and protect lives and property.

Re-blocking is seen as one such measure (Basson 2019; Brown-Luthango et al. 2016; Mtani & Mbuya 2018; Rush et al. 2020; Tsebe 2020). Although re-blocking aims to improve settlements in a range of ways, fire-risk reduction is an important objective of the City of Cape Town’s re-blocking policy, adopted in 2013 (City of Cape Town 2012), and it is often implemented after extensive fires where large numbers of houses are destroyed. By de-densifying informal settlements, improving road access, upgrading services and improving the quality of houses, re-blocking can in principle reduce fire risk (Spinardi, Cooper-Knock & Rush 2020). In practice, although beneficial in the short term, the re-blocked informal settlements often re-densify (Walls 2018), undoing the spatial changes that could reduce fire risk. Understanding why settlements re-densify can help to improve the longevity of interventions and strengthen fire risk reduction in informal environments, in South Africa and comparable settings elsewhere.

The research in this article examines re-blocking through case studies of two informal settlements, namely Mshini Wam and Sondela, in Cape Town. The aim of the study was to explore informal settlement dwellers’ perspectives on re-blocking and the factors influencing the success of interventions. The purpose was not to investigate the successfulness of re-blocking as a fire reduction strategy, but instead, given the authorities’ adoption of re-blocking as a fire risk reduction in Cape Town, people’s experiences and attitudes towards re-blocking and the dynamics affecting the longevity of spatial changes. It begins by briefly describing the purpose and process of re-blocking and the benefits and the challenges associated with implementing re-blocking projects. It then introduces the case study sites and presents findings on the success of the projects, people’s understanding of re-blocking and reasons for ‘re-densification’. It then discusses the findings with respect to the literature on the implementation of re-blocking and draws out the lessons learned.

### The purpose and practice of re-blocking

Re-blocking is a form of informal settlement upgrading (Community Organisation Resource Centre [CORC] 2013; Hennings et al. n.d.; Worcester Polytechnic Institute [WPI] 2012). It aims to reconfigure the spatial layout of informal settlements to reduce housing density and create space for roads and basic service infrastructure. In addition to creating space, re-blocking projects usually include improving access to basic services, including water and sanitation infrastructure and electricity (Gontsana 2016), as well as improvements to the materials used for dwellings.

This can reduce fire risk in several ways. Creating spaces between houses can help decrease the density of informal settlements, slowing the spread of fires. By creating roads, re-blocking can improve access for emergency personnel and vehicles and provide pathways for evacuations (Spinardi et al. 2020). Spaces can also serve as fire breaks and reduce the spread of fires between adjacent settlements (Spinardi et al. 2020; Walls 2020). The installation of water infrastructure helps communities and authorities to more easily and effectively fight fires, while access to safe electricity connections lowers the need for dangerous energy sources and reduces exposure to fires (Spinardi et al. 2020). Rebuilding dwellings with more fire-resistant materials can also help to reduce the likelihood and spread of fires (Basson 2019; Brelsford et al. 2018; Satterthwaite et al. 2020; Visagie & Turok 2020).

Re-blocking is envisaged as a community-driven process, which allows affected people to identify their own challenges, plan and design the settlement layout to best suit their needs, with the authorities playing a supportive role (Zikalala 2019). The re-blocking methodology was developed by Slum Dwellers International (SDI) and is implemented in Cape Town through a collaborative partnership between the Informal Settlement Network (ISN), which is affiliated with SDI, the CORC and the City of Cape Town (Hennings et al. n.d.; Kiefer & Ranganathan 2018). The ISN, a community-based organisation (CBO) comprised informal settlement residents from across South Africa, helps to identify and mobilise communities, and CORC provides financial and technical support to both the partners and community. The City of Cape Town also assists financially, provides shack materials and is responsible for the installation of services, such as water taps and toilets (Hennings et al. n.d.).

Although there are limited examples of re-blocking being adopted specifically as a fire-risk reduction measure internationally, it has been used in the upgrading of informal settlements. Although not labelled as re-blocking per se, the Baan Mankong programme, launched in 2003 in Thailand, represents an example of large-scale *in situ* upgrading. It illustrates that re-blocking can be scaled nationally as part of a broader upgrading strategy, balancing improved safety (including reduced fire vulnerability) with tenure security and community ownership (CODI n.d.). Upgrading projects have also been implemented in Pune, in India (SDI 2013); Kotabaru, Indonesia (Caesarina 2023) and in Brazil (Magalhaes & Di Villaro 2012).

**FIGURE 1:**
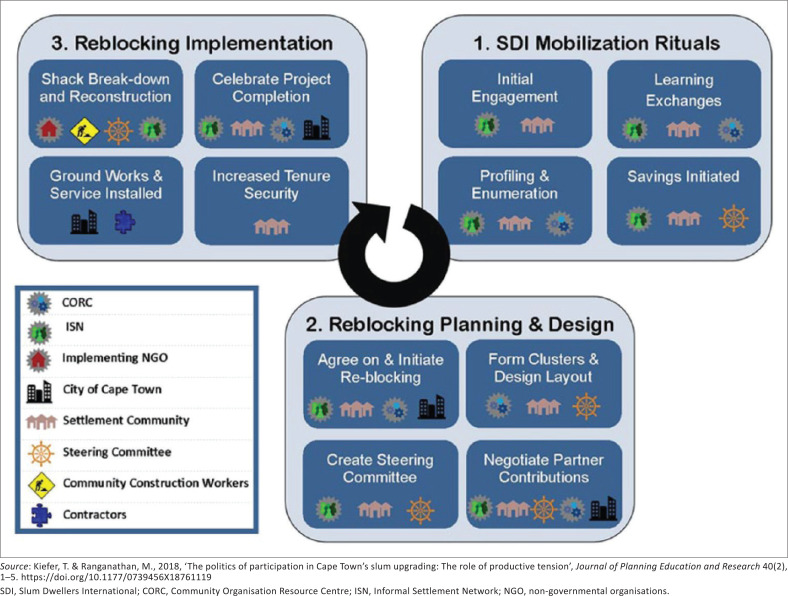
A summary of the re-blocking process.

As shown in Figure 1, the generic re-blocking process involves three phases, spanning mobilisation, planning, design and implementation (Kiefer & Ranganathan 2018). The first involves mobilising the community and community-level funding, building trust and relationships, as well as profiling and enumerating the settlement to determine population numbers and needs. The second stage involves collaboratively planning implementation, as well as determining partner contributions, and establishing oversight mechanisms. This phase includes the participatory mapping of settlements and residents co-designing and agreeing on the re-blocking layout. The third stage is the implementation phase, where shacks are broken down and then rebuilt, and electricity and water and sanitation infrastructure are installed. Residents are also granted occupancy rights, providing a degree of tenure security (Kiefer & Ranganathan 2018).

### Benefits and challenges of re-blocking

Literature identifies a range of benefits. These including generating safer living environments (Brown-Luthango, Reyes & Gubevu 2017; Caesarina 2023; CODI n.d.; Magalhaes & Di Villaro 2012; Mitra et al. 2017; SDI 2012; Zikalala 2019), increasing tenure security (CODI n.d.; Magalhaes & Di Villaro 2012; SDI 2012), improving quality of life (Brown-Luthango et al. 2017; Tsebe 2020) and fostering community solidarity (SDI South African Alliance 2013). The collaborative process can also create a sense of pride and ownership, provide job opportunities, create a sustainable change (Hennings et al. n.d.), as well as enhance community organisation and provide participants with a voice in urban governance (Caesarina 2023; CODI n.d.; Magalhaes & Di Villaro 2012; SDI 2012). Facilitated well, re-blocking interventions can also help to sensitise people to the dangers of densification and the need for space to improve access, not only for emergency services but also public amenities such as water taps and toilet facilities (Tshabalala & Mxobo 2014).

Research and experience also suggest a range of challenges associated with re-blocking. These include the cost and complexity of re-blocking, institutional fragmentation and silos that hamper joined up decision-making and complicate funding arrangements, as well as limited bureaucratic and financial support from government institutions (Brelsford et al. 2018; Daniels et al. 2016; Hendler & Fieuw 2018; Hennings et al. n.d.; Kiefer & Ranganathan 2018; Zikalala 2019). Unclear roles and responsibilities can delay projects and reduce their effectiveness. Resistance from residents, ‘shack farming’ by landlords and differing expectations can also complicate the process (Brelsford et al. 2018; Walls 2020). A lack of trust, particularly between informal communities and governments and politicians, can make collaboration challenging, while role-players’ different backgrounds, social divides and language barriers can complicate the trust building process (Hennings et al. n.d.). The logistics of upgrading and balancing the technical approach and standards adopted by municipalities with the abilities and work style of communities can also be difficult (Hennings et al. n.d.).

These problems are not unique to South Africa, with the upgrading projects in Thailand, India, Indonesia and Brazil identifying similar issues. These include disputes over allocation of units and concerns over losing tenure (CODI n.d.); residents’ reluctance to relocate (Caesarina 2023; CODI n.d.; Magalhaes & Di Villaro 2012); insufficient funding and resource constraints (Caesarina 2023; CODI n.d.; Magalhaes & Di Villaro 2012; SDI 2012); weak governance and planning support (Caesarina 2023; CODI n.d.); political discontinuity and fragmentation between municipal bodies, housing boards and non-governmental organisations (NGOs) implementing projects (Magalhaes & Di Villaro 2012; SDI 2012) and high settlement densities and terrain, which made reworking settlements challenging (Caesarina 2023; CODI n.d.; Magalhaes & Di Villaro 2012; SDI 2012). These challenges underline the need for strong community participation, reliable financing and supportive institutional frameworks for re-blocking to work as an effective risk reduction strategy. In Brazil (Magalhaes & Di Villaro 2012), analysis also identified issues such as elite capture of projects and differential benefits across different neighbourhoods.

A participatory approach should help to improve the successfulness of interventions (Dodman & Mitlin 2013; Walls et al. 2020). Participation allows beneficiaries to shape projects that impact their lives. This not only helps to generate buy-in but ensures that interventions are acceptable, appropriate and sustainable (Dodman & Mitlin 2013; Maganadisa, Letsoko & Pretorius 2021; Mubita, Libati & Mulonda 2017; Nkombi & Wentink 2022; Parikh et al. 2020; Scolobig et al. 2015; Zikalala 2019). They can build trust and foster productive consultation and negotiation between stakeholders (Klug & Vawda 2009; Maganadisa et al. 2021; Mubita et al. 2017; Parikh et al. 2020). They can also help to make projects more equitable and community oriented (Meredith & MacDonald 2017). Participatory approaches are also a cornerstone of disaster risk reduction. The United Nations Office on Disaster Risk Reduction (UNDRR), for instance, argues that communities possess vital local knowledge and must be involved in all risk reduction activities (UNDRR n.d.).

However, participatory approaches are challenging to implement. Communities are diverse and heterogeneous, encompassing a range of often competing perspectives, needs and agendas (White 1996). Processes can be dominated by the powerful (Chambers 1994; Haynes 2020; Mubita et al. 2017; Rigon 2014) and may fail to capture the views of all community members, leading to agreements that do not reflect true consensus (Maganadisa et al. 2021). In these cases, and where policies and projects are poorly articulated or communicated, interventions may not meet beneficiaries’ needs and expectations (Brown-Luthango et al. 2017) leading to unhappiness and people disengaging (Bayat 2000; Brelsford et al. 2018; Khan & Wallis 2015; Tshabalala & Mxobo 2014). Although some argue that tension can promote mutual learning and help to build momentum and collaboration (Kiefer & Ranganathan 2018), inadequate trust and competing agendas can discourage participation and create conflict that slows or jeopardises processes (Hendler & Fieuw 2018; Nkombi & Wentink 2022; Parikh et al. 2020; Zikalala 2019). Processes also can place an additional burden on local communities, especially where adequate institutional support is not provided (Haynes 2020). In addition, governmental role-players, in particular, often lack the skills and knowledge to engage in participatory processes (Parikh et al. 2020; Zikalala 2019). Interventions can also be tokenistic (Haynes 2020) and can treat participation as a consultative process rather than a genuinely collaborative one, resulting in decisions that overlook community perspectives and needs (Maganadisa et al. 2021; White 1996).

Against this background, the research sought to explore the dynamics impacting the successfulness of re-blocking in the case study areas, including people’s experiences, the inclusiveness of the participatory process and how re-blocking could be strengthened in the context of fire-risk reduction.

## Research design and methods

The research adopted a qualitative research approach that combined in-depth key-informant interviews and focus groups in the re-blocked settlements.

### Site selection, sampling and data collection

Key informants were selected purposively. Interviewees were intentionally selected for their skills, knowledge and experience in the process of re-blocking, fire-risk reduction and informal settlement upgrading. A total of eight in-depth semi-structured interviews were conducted. Three in-depth interviews were conducted with officials from the City of Cape Town’s Disaster Risk Management Centre (two) and Informal Settlements Department, respectively (one). Another interview was conducted with a representative from CORC and ISN, which facilitate re-blocking in Cape Town. In addition, two interviews were conducted with community leaders in Mshini Wam and three in Sondela. Each interview lasted approximately 1 h.

The interviews explored the rationale for re-blocking and the associated challenges, as well as the basis on which settlements qualify for re-blocking, and how they go about engaging targeted communities to obtain community buy-in. Community leaders were interviewed to obtain their perspectives on re-blocking as beneficiaries, as well as to understand the settlements’ history and context and their views on the re-blocking interventions.

The two communities were chosen with the assistance of CORC and ISN. Mshini Wam was selected because it was the earliest example of re-blocking in Cape Town and hence provided a good case study to explore re-densification. Sondela was more recently re-blocked and provided an opportunity to examine learning in the process over time. Three focus groups were run in each community. One was conducted with influential people in the community, one with landlords and one with other community members. The selection of focus group participants was based on those who attended community meetings concerning re-blocking, were involved in the (re)design of their settlement and had lived in the settlement prior to re-blocking. The rationale was for residents to provide contextual information on their settlements, their expectations and factors affecting the success of the interventions, as well as why people often build in cleared spaces. The focus groups were conducted in Xhosa, the predominant language in the settlements. Each focus group included 12 participants and lasted approximately 2 h. In these focus groups, participants were able to share their perceptions of re-blocking as a fire-risk strategy, as well as their experiences during implementation and afterwards. All in all, 84 people participated in the study.

### Data analysis

The interviews and focus groups were recorded electronically with participants’ permission. The recordings were transcribed, and the data analysed thematically to identify topics and issues emerging from the data.

### Study’s limitations

The methodology adopted has limitations. The public nature of focus group discussions may have led to homogenisation of responses and people may not have surfaced divergent or negative opinions, especially with respect to other people’s actions or their own. More confidential tools such as interviews might have better identified such issues. Another limitation is that Sondela had been recently re-blocked, allowing insufficient time to determine the extent of re-densification. The researcher chose this recent case because accessing some settlements proved difficult, but it would have been preferable to conduct the research on an older project. Nonetheless, the research in Sondela highlights important issues regarding engagement and people’s experience of re-blocking.

### The case study sites

Mshini Wam was chosen as a case study because it was among the first informal settlements to undergo re-blocking. The settlement was re-blocked in 2012 (Hennings et al. n.d.). Sondela is a newer settlement, where implementers were able to draw on the experiences of settlements such as Mshini Wam. The settlement is an example of ‘relocation re-blocking’ or greenfield re-blocking, with residents moved to the new site in October 2023.

Mshini Wam forms part of Joe Slovo Park in Milnerton. Mshini Wam is 13 km away from Cape Town’s city centre and lies to the northeast of the city. A household enumeration survey conducted in 2012, just prior to the settlement being re-blocked, showed that there were 497 people living in Mshini Wam at the time, comprising 250 shacks (Tshabalala & Mxobo 2014).

The settlement was selected for re-blocking owing to the frequency and impact of fires. Residents approached the City of Cape Town following several fires, with the City introducing community leaders to the ISN. The settlement was re-blocked in 2012. The spatial plan created clusters of between 8 and 10 shacks. An elected community team and an architect from CORC developed the spatial layout, with the plan approved by the City of Cape Town in 2012 (WPI 2012). The project was funded by the community themselves, through their saving schemes, and CORC also assisted financially. The City of Cape Town supplied residents with roofing material, sand filling, crushing stones and compacting machinery (Bolnick & Bradlows 2012).

**FIGURE 2:**
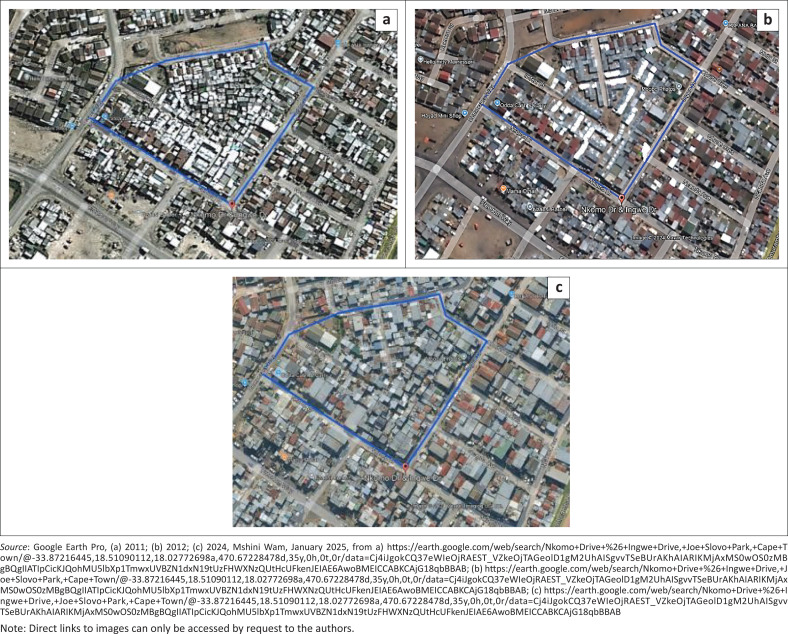
Re-blocking and re-densification in Mshini Wam.

The project explicitly sought to address fire risk by rebuilding dwellings using fire resistant materials and designing spaces to allow large emergency vehicles to access the settlement (Henning et al. n.d.). Figure 2 shows Mshini Wam (outlined in blue) prior to re-blocking in 2012 (2a), immediately after the re-blocking (2b) and the settlement in 2024 (2c). As illustrated in the images, the re-blocking successfully served to de-densify the settlement initially, but it has to a large degree re-densified in the years since (2c).

The other settlement, Sondela, is in Mfuleni on the Cape Flats, to the southeast of the city. Sondela is 21.8 km away from Cape Town. Sondela was created when people living in informal ‘backyard’ dwellings in Mfuleni invaded land owned by the City of Cape Town. The settlement initially comprised 200 individuals, but the population increased over the years (Community Leader [Sondela] pers. comm., 2023). In 2016, the City of Cape Town identified a piece of vacant land close to the original settlement, with the re-blocking process starting in 2019 (Community Leader [Sondela] pers. comm., 2023). The settlement could not be upgraded *in situ* because its location under electricity power lines was considered unsafe and prevented the City of Cape Town from delivering services. The City of Cape Town views this re-blocked settlement as interim housing, the long-term plan being to upgrade dwellings to formal subsidised housing units in the future – although no time period for this has been provided (Obese 2023).

As in Mshini Wam, CORC ran engagement workshops and provided financial support, while the ISN helped to facilitate the process. Community members also contributed financially towards the project, with co-funding from the City of Town. The City of Cape Town was responsible for providing all the underground services such as the sewers and water network, above-ground infrastructure and electrification (City of Cape Town 2023).

**FIGURE 3:**
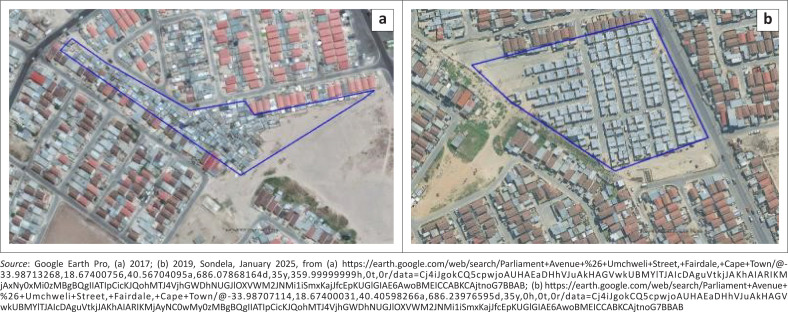
(a) Sondela prior to re-blocking in 2017; (b) Sondela after re-blocking, as of 2024.

The physical layout of the settlement was determined by the community members working together with CORC, after they were taken to Mshini Wam to see the results there. Community Organisation Resource Centre submitted the plan to the City of Cape Town’s Informal Settlements Department, which allocated a plot size of 18 m^2^ for each household. Figure 3a, shows the layout of the settlement on the original site prior to re-blocking and Figure 3b the settlement following re-blocking.

There was an emphasis on participation in both projects. The representative from CORC explained that community engagement processes involve several sessions in the hope of capturing as diverse a range of perspectives as possible. These include leadership engagements, community general meetings and settlement cluster meetings. This approach provides people with an opportunity to participate and contribute to the process. However, the representative found that some people do not attend engagements, only involving themselves when their units are re-positioned or resized.

### Ethical considerations

Ethical clearance to conduct this study was obtained from the from Stellenbosch University’s Human Research (Humanities) Ethics Committee (No. REC: SBE-2023-27489). The project also received ethical approval from the City of Cape Town (Reference number PSRR-1038). Participants signed written consent forms before participating in the interview or focus group. In keeping with ethical approval standards, interviews were conducted one-on-one in a private setting to protect confidentiality, with focus group participants undertaking not to share any of the discussions with others. Data were stored securely, and only the lead researcher had access to the data.

## Results

### The successfulness of the interventions

Re-blocking in Mshini Wam successfully de-densified the settlement, but re-densification since 2012 suggests that the intervention has not been optimally successful in the long term. Despite this ostensible ‘failure’ of the project, participants in Mshini Wam felt the intervention was successful owing to the provision of basic services, improved road access and electricity. Participants from Sondela were also mostly positive for the same reasons, but as discussed later, some felt excluded and that the process had been rushed. Some also felt frustrated by the perceived delays in upgrading to formal housing and felt that this would undermine re-blocking. As one participant argued:

‘[*T*]hey will build on cleared spaces because they run out of patience, as re-blocking is introduced to them as an interim phase but lasts longer than the expected stay they were told about.’ (Other community member, Sondela, 48, Male)

### People’s understanding of the purpose of re-blocking

The findings suggest that while officials viewed re-blocking both as an opportunity to improve service delivery and as a fire risk reduction measure, beneficiaries viewed it primarily in terms of service delivery. One respondent explained, re-blocking is ‘a mechanism used by the City of Cape Town to provide services to the community’ (Community member, Sondela, 35, Male), while another reported that it ‘is also to introduce safety to the settlements’ (Other community member, Sondela, 33, Female). However, one member from Sondela also commented:

‘I could not understand the process of re-blocking. First, I thought the City of Cape Town was moving us for better housing options but instead we were moved to much smaller shacks than the ones we previously owned. I do not understand even now. Maybe the purpose was for services because people say to me [*sic*]. I do not understand. Because even when we attended meetings with our community leaders, we were told that this is an interim project and that we will be given houses from here.’ (Other community member, Sondela, 40, Male)

People’s expectations included being offered water, obtaining electricity of their own, sanitation infrastructure and better living conditions. Some respondents in Mshini Wam understood the purpose of re-blocking to be making the community safer although the majority of participants in both settlements saw safety in terms of crime and violence as opposed to fire risk. Residents of Mshini Wam found that before re-blocking they were afraid to walk in the neighbourhood at night but after re-blocking they felt safe because they could walk around the community and could see people from afar.

### Factors determining the success of projects

Both officials and community members agreed that effective community engagement, transparency and communication were essential for success. As one participant observed:

‘[*A*] successful re-blocking project is achieved when the government recognises and works hand in hand with community leaders, community members, and community organisations.’ (Community Leader, Sondela, 38, Male)

However, although ISN and CORC undertook to make the process participatory, some residents felt pressured. Many felt that the process was rushed, with one focus group participant arguing that:

‘… it was as if the authorities just wanted a yes from the re-blocking [*sic*] so they can start the process, without fully engaging the community.’ (Community member, Sondela, 35, Female)

Others felt left out, highlighting that the trip to Mshini Wam was only attended by community leaders.

Participants also flagged that re-blocked settlements did not cater for social amenities, such as churches, playgrounds and other recreational areas, which they viewed as important in guaranteeing the long-term success of re-blocking initiatives. A respondent argued that:

‘In the old informal settlement they used to stay at, they had a gym facility which the community helped to build but now that they are relocated to a re-blocked land such facilities are not accommodated and that they are being told by City of Cape Town official to not extend or build anything on the newly re-blocked land.’ (Community Leader, Sondela, 36, Male)

From CORC’s perspective, funding was an issue. They found that the success of re-blocking is hindered by insufficient municipal funding. They maintained that although many people are receptive to the idea of crowdfunding, some residents are sceptical or lack the financial means to contribute. This causes delays and can result in residents disengaging. They observed that expecting poor community members to co-fund upgrading is unrealistic and creates problems, and that the City of Cape Town should set aside a budget to implement re-blocking projects. They also felt that governmental partners struggle to understand participatory processes and to balance technical requirements and the needs identified by communities.

### Reasons for re-densification

People provided a range of reasons for people building in spaces cleared by re-blocking. Both officials and community members agreed that the most common reason was that the houses provided are too small to cater for households’ space requirements. One member explained:

‘The newly built shack by the City of Cape Town does not accommodate my family. Therefore, to accommodate my family I need to utilize the unused space in front of this shack to extend and build an additional room, because I stay with my husband and seven children. I had to separate my family because not all of us could sleep in the newly built shack. My older children are sleeping elsewhere as I had to arrange a place for them to sleep, they only come home to eat then at night they must go to the place I arranged for them.’ (Other community member, Sondela, 30, Female)

Other reasons included both opportunism and the use of rebuilding as a tool for pressuring the City to improve conditions. In Mshini Wam, a participant noticed that she just saw an opportunity to make her dwelling larger:

‘The reason I built on the cleared space was that my house had caught fire, so in rebuilding my settlement I saw the opportunity to extend it.’ (Other community member, Mshini Wam, 30, Female)

This was not seen as a source of conflict or friction within the community, but it did encourage other members to build in cleared spaces. Another focus group respondent in Mshini Wam argued that in many cases:

‘Community members extend their homes to draw the attention of the City of Cape Town, expecting it will encourage official intervention or support. The municipal intervention can range from infrastructure upgrades, housing improvements, the establishment of health and social services, and legal intervention.’ (Other community member, Mshini Wam, 33, Male)

Although shack farming was not flagged as a significant issue, landlords reported feeling that their needs were not considered. One participant observed, ‘So for us, re-blocking does not consider landlords even [*in the allocation of*] the space, landlords are not accommodated’ (Landlord, Mshini Wam, 45, Male). They argued that this impacts household livelihoods ‘because we are unemployed and through renting space we can provide for our families’ (Landlord, Mshini Wam, 2023). Another from the same group added:

‘I also think that re-blocking process does not consider the existing economy on [sic] the community, or rather let me say the daily sustenance of people, because if they did they would consider providing something for those who will be affected.’ (Landlord, Mshini Wam, 53, Male)

According to CORC, ownership is one of the main dynamics affecting the re-blocking process as the people staying in the settlements are often tenants (CORC 2023). This impacts re-blocking because tenants may require landlords’ approval for alterations, slowing the process and leading to disagreements with the property owner. They maintained that tenants also often engage less in community efforts because they do not envisage living in the settlement in long term.

Some participants suggested that formalising roads in re-blocked settlements can play a key role in preventing re-densification and act as a deterrent to building in cleared spaces. Some participants found that obtaining occupancy rights helped to motivate action by dwelling owners; when residents feel secure in their tenure, reflected through the allocation of stand numbers, they are more likely to invest in their homes and support the upgrading process.

## Discussion

### Participants’ expectations of re-blocking

The findings suggest some divergence between officials’ and beneficiaries’ expectations. While City officials viewed re-blocking as a way to both improve service delivery and reduce fire risk, residents focused on re-blocking as a measure to improve their quality of life. Expectations included access to services, a safe and secure environment and poverty alleviation. In Mshini Wam, people felt that the interventions were successful despite re-densification, primarily because of the provision of services. Although some saw re-blocking as a fire-risk reduction strategy, this was not a common measure of success. It is possible that promoting re-blocking as a fire-risk reduction strategy could reduce re-densification although other factors, such as people’s need for space, may override concerns around fires.

One of the key benefits associated with re-blocking is regularising settlements and improving security of tenure (Brelsford et al. 2018; CODI n.d.; Magalhaes & Di Villaro 2012; SDI 2012). The findings suggest that for residents this was among the most important measure of success. The findings also suggest that occupancy rights may encourage investment in dwellings and reduce re-densification. The literature suggests that security of tenure reduces people’s fears of eviction and fosters home improvements (CODI n.d.; Magalhaes & Di Villaro 2012; Tshabalala & Mxobo 2014). Applied to the cases studies, a stronger sense of ownership could conceivably promote improvements that reduce risk, although if re-blocking is seen as a path to tenure security, this could also result in in-migration and the growth of settlements, especially where upgrading takes time.

### Reasons for re-densifying

Participants highlighted several reasons for re-densifying. Unlike previous studies (e.g. Brelsford et al. 2018; Walls 2020), shack-farming did not appear to be a major contributor in the case study areas, but space was. Building into the spaces cleared is inevitable where housing does not meet beneficiaries’ needs (Brown-Luthango et al. 2017; Isandla Institute 2019; Khan & Wallis 2015). The size of dwellings was often considered too small to accommodate larger families and possessions or to accommodate the growth of households over time. The findings also suggest that in-filling can be a form of protest and was sometimes perceived as a tool for forcing the municipality to address service delivery needs. Although not a cause of re-densification, participants were unhappy about the exclusion of social amenities such as church and recreational facilities, highlighting the importance of not only focusing on housing but also, as Brown-Luthango et al. (2017) observe, social infrastructure that speaks to people’s relationships, sense of belonging, community and well-being.

The study did not identify many of the institutional problems associated with re-blocking, but in line with some literature (Brelsford et al. 2018; Caesarina 2023; CODI n.d.; Daniels et al. 2016; Kiefer & Ranganathan 2018; Magalhaes & Di Villaro 2012; SDI 2012), the study suggests that finding the money to implement projects can be an obstacle. The research suggests that sufficient funding from the government for the re-blocking process is crucial as communities often do not have the financial resources to co-fund projects.

### The importance of meaningful engagement

Literature highlights the importance of participation in generating buy-in and improving projects’ responsiveness and successfulness (CODI n.d.; Dodman & Mitlin 2013; Maganadisa et al. 2021; Mubita et al. 2017; Scolobig et al. 2015; Twigg 1999), building trust (Klug & Vawda 2009; Maganadisa et al. 2021; SDI 2012) and avoiding misunderstandings (Bayat 2000; Khan & Wallis 2015; Magalhaes & Di Villaro 2012; Tshabalala & Mxobo 2014). Officials, CORC and community members underscored the importance of engaging community members in re-blocking. However, the findings also highlight the challenges associated with participatory processes, particularly with respect to the reach and successfulness of engagement. While most of those spoken to felt that they had been included, some focus group participants in Sondela felt that processes had been rushed, and that their needs were not represented or integrated authentically into the planning and implementation. This, as found elsewhere (Bayat 2000; Brown-Luthango et al. 2017; Khan & Wallis 2015; Tshabalala & Mxobo 2014), had led to dissatisfaction with the results. In heterogeneous communities, it may be impossible to address everyone’s needs and expectations (White 1996), especially where people do not participate in engagement platforms, but the findings reiterate the importance of allowing adequate time and space for deep engagement, as well as ensuring that processes are as inclusive as possible.

## Conclusion

The use of dangerous energy sources and flammable housing materials increase the likelihood of fires in informal settlements, while high residential densities, poor access and inadequate water infrastructure help to increase the spread of fires. In this context, the authorities in Cape Town have identified re-blocking as an important fire-risk reduction measure. Re-blocking interventions, implemented in settlements such as Mshini Wam and Sondela, aim to improve fire safety (and living standards) in informal settlements, by creating roads, increasing the space between dwellings and installing water and electricity infrastructure. Increasing the space between dwellings settlements and improving access are central to re-blocking as a fire-risk reduction measure, but experience suggests that settlements often re-densify. Understanding why settlements re-densify can help to improve the longevity of re-blocking initiatives and any benefits with respect to reducing fire risk.

The findings of this study suggest that re-densification occurs for a range of reasons. People in the two case study settlements did not always understand the purpose of re-blocking, and beneficiaries’ expectations differed from officials’, with community members interpreting re-blocking primarily in terms of improving access to services and quality of life as opposed to fire-risk reduction. Despite implementers’ commitment to participation, some felt excluded, confused and unhappy with the outcomes. Although some re-densification was opportunistic, participants reported that people primarily rebuild shacks in cleared spaces because houses are too small and do not cater for their needs. Rebuilding may also be form of protest or a tool for claiming perceived rights. Being free from evictions is also a factor in people building in cleared spaces.

The findings suggest that the longevity and success of projects depend critically on ensuring projects meet beneficiaries’ needs and expectations. The findings underscore that deep and inclusive community engagement is essential to surface and capture these diverse needs, including those related to social amenities to achieve longer-lasting results and the intended benefits for fire-risk reduction.
